# Multi-Functionalized Self-Assembling Peptides as Reproducible 3D Cell Culture Systems Enabling Differentiation and Survival of Various Human Neural Stem Cell Lines

**DOI:** 10.3389/fnins.2020.00413

**Published:** 2020-05-05

**Authors:** Amanda Marchini, Chiara Favoino, Fabrizio Gelain

**Affiliations:** ^1^Tissue Engineering Unit, Institute for Stem Cell Biology, Regenerative Medicine and Innovative Therapies-ISBReMIT, Fondazione IRCSS Casa Sollievo della Sofferenza, San Giovanni Rotondo, Italy; ^2^Center for Nanomedicine and Tissue Engineering, ASST Grande Ospedale Metropolitano Niguarda, Milan, Italy; ^3^Department of Biotechnology and Biosciences, University of Milan-Bicocca, Milan, Italy

**Keywords:** human neural stem cells, self-assembling peptides, three-dimensional cell cultures, neuronal differentiation, multi-functionalized scaffold, nervous tissue engineering

## Abstract

Neural stem cells-based therapies have shown great potential for central nervous system regeneration, with three-dimensional (3D) culture systems representing a key technique for tissue engineering applications, as well as disease modeling and drug screenings. Self-assembling peptides (SAPs), providing biomimetic synthetic micro-environments regulating cellular functionality and tissue repair, constitute a suitable tool for the production of complex tissue-like structures *in vitro*. However, one of the most important drawbacks in 3D cultures, obtained via animal-derived substrates and serum-rich media, is the reproducibility and tunability of a standardized methodology capable to coax neural differentiation of different human cell lines. In this work we cultured four distinct human neural stem cell (hNSC) lines in 3D synthetic multifunctionalized hydrogel (named HYDROSAP) for up to 6 weeks. Three-dimensional cultures of differentiating hNSCs exhibited a progressive differentiation and maturation over time. All hNSCs-derived neurons in 3D culture system exhibited randomly organized entangled networks with increasing expression of GABAergic and glutamatergic phenotypes and presence of cholinergic ones. Oligodendrocytes formed insulating myelin sheaths positive for myelin basic protein (MBP). In summary, results demonstrated a successfully standardized and reproducible 3D cell culture system for hNSC differentiation and maturation in serum-free conditions useful for future therapies.

## Introduction

Conventional *in vitro* neuronal models rely on cell growth in 2D platform that are a poor representation of cell behavior *in vivo*. In contrast, 3D cell culturing, by more closely mimicking the cellular microenvironments, aims to re-create *in vitro* similar chemical and mechanical properties of native central nervous system (CNS) tissue directing specific cell–substrate interactions and controlling cell attachment, migration, survival, and differentiation toward specific cellular phenotypes (Duval et al., 2017).

Hydrogel-based biomaterials are an excellent platform for 3D cultures because of their biocompatibility, elasticity, adjustable mechanical properties, and the ability to mimic native extracellular matrix (ECM). They have been widely used in biomedical researches, ranging from tissue engineering (Christman, 2019) and regenerative medicine (Cembran et al., 2020) to drug delivery (Narayanaswamy and Torchilin, 2019) and microfluidic devices (Alessandri et al., 2016; Natividad-Diaz et al., 2019). Hydrogels provide a tissue-like environment thanks to a network of exogenous proteins/polymer derived from natural (collagen, laminin, alginate, hyaluronic acid) or synthetic source (such as PEG and polyacrylamide), that can facilitate the differentiation of stem cells and the formation and maturation of neuronal networks in a 3D fashion. Among natural materials, Collagen type I was recently used to reproduce a long-term 3D neuronal culture (Lam et al., 2020); similarly, 3D expansion and long-term differentiation of neural precursor cells were supported by a tailored synthetic poly(ethylene glycol) diacrylate-crosslinked porous polymeric biomaterial (Murphy et al., 2020). Additionally, Collagen has been used as “patches” for *in vivo* transplantation approaches for the regeneration of injured and/or non-functional tissues. Indeed, *in vitro* 3D hydrogel cultures provide new tools to direct stem cell differentiation into defined phenotypes prior transplantation (Lai et al., 2018).

Matrigel substrate (or Cultrex) offer many advantages for studying cell migration and differentiation, angiogenesis and tumor development: but their animal origin, undefined composition and batch-to-batch variability (in terms of mechanical and biochemical properties) are significant drawbacks (Benton et al., 2014). Lancaster et al. (2013) developed a human pluripotent stem cell-derived 3D organoid culture system that display *in vitro* self-organization of brain regions and recapitulate features of human cortical development. This is an exceptional tool to study principles of developmental biology (Renner et al., 2017) and to reproduce human diseases difficult to replicate *in vivo* experiments, but with limited translational potential.

Conversely, self-assembling peptides (SAPs), with nanofibrous networks mimicking ECM, have shown a remarkable potential because of their synthetic source, biocompatibility and biomimetic properties (Pugliese and Gelain, 2017; Li et al., 2019). By adding functional motifs, functionalized or multifunctionalized SAPs can be customized to promote neurite outgrowth, neuron differentiation, cell adhesion and so on, for different applications and/or therapeutic treatments (Pugliese et al., 2018b).

We previously developed a multifunctionalized SAPs scaffold enabling the 3D culturing of human neural stem cells (hNSCs) by guiding cell growth and generating mature and electrically active neurons. Examination of the cell-embedded scaffolds showed that hNSCs were viable in long-term cell cultures and, after pre-differentiation *in vitro* for 6 weeks, neuroregenerative potential was testified by showing decreased astrogliosis, low immune response, high percentage of neuronal markers, hNSCs engraftment and improved behavioral recovery in rat spinal cord injury (Marchini et al., 2019).

However, a feasible translational therapy should take into account result reproducibility, or, conversely, a careful matching between differentiated progeny vs injury requirements, and a better understanding of the time-dependent maturation of these densely cultured 3D patches.

Here, we further developed and validated a reproducible and standardized protocol to obtain a serum-free hNSC-HYDROSAP. We analyzed four distinct lines of good manufacturing practice (GMP) protocol-grade (GMP-grade) hNSCs at 1 day, 1 week, and 6 weeks *in vitro* (1DIV, 1WIV, and 6WIV, respectively). SAPs hydrogel (pureHYDROSAP) was used to direct cell differentiation and maturation by providing multi-functionalized 3D microenvironments. As a reference gold-standard, hNSCs were cultured on 2D coatings of CULTREX substrate and compared with 3D HYDROSAP-based cultures at 1WIV: results showed decreased percentages GFAP+ cells with increased presence of Nestin+ progenitor cells.

Instead, in long-term 3D HYDROSAP cell cultures cell proliferation and presence of Nestin+ cells decreased over time, counterbalanced by similar concomitant increments of GABAergic and glutamatergic neuronal phenotypes. On the other hand, cell lines showed peculiar levels of expression of neuronal markers such as MAP2, GAP43, and SMI31. Results demonstrated the formation of entangled and randomly organized neuronal networks at 6WIV and excellent neuronal differentiation highlighted by robust expression of GABAergic, glutamatergic and cholinergic neurons and mature oligodendrocytes expressing myelin basic protein (MBP) marker.

## Materials and Methods

### Ethics Statement

Human neural stem cells were produced according to GMP protocols, as dictated by the European Medical Agency (EMA) guidelines. The tissue collection procedure, the cell factory, the production procedure and the cell validation criteria received formal approval and certification by the appropriate regulatory body, namely the Agenzia Italiana del Farmaco, protocol number aM 101/2010 (updated in 2018 after AIFA inspection to number aM 54/2018). Human fetal brain tissue specimens, all derived from the forebrain, were routinely collected from spontaneous miscarriages at gestational ages greater than the 8th post-conceptional week. They were immediately dissected and used to generate hNSC lines under sterile conditions. Tissue procurement was approved by the ethical committee of Umbria (Ethical Committee of Umbria’s Health Institutes, CEAS) and was possible exclusively upon the mother giving informed, written consent. Also, specimen collection and medical procedures were in full accord with the Helsinki declaration (WMA Declaration of Helsinki – Ethical Principles for Medical Research Involving Human Subjects).

### Synthesis and Purification of PureHYDROSAP

PureHYDROSAP was prepared as previously described (Marchini et al., 2019). Briefly, it is composed by a linear SAP, two differently functionalized and a branched SAP, all sharing the same (LDLK)3 self-assembling backbone (Gelain et al., 2012; Caprini et al., 2013; Pugliese et al., 2018a). Peptides were synthesized by solid-phase Fmoc-based chemistry on Rink amide 4-methyl-benzhydrylamine resin (0.5 mmol g^–1^ substitution) with the Liberty-Discovery (CEM) microwave automated synthesizer. The side chains removal and cleavage were performed with TFA:TIS:H2O (95:2.5:2.5) cocktail. Cleaved peptides were precipitated using cold ethyl ether and then lyophilized (Labconco) and the resulting raw peptides were purified by a Waters binary HPLC (>95%). The molecular weight of purified peptide was confirmed via single quadrupole mass detection (Waters LC-MS Alliance-3100). Purified peptides powder was subsequently dissolved in 0.1 M HCl in order to remove the presence of possible TFA salts.

### hNSCs Culture and hNSC-HYDROSAP Preparation

**FIGURE 1:**
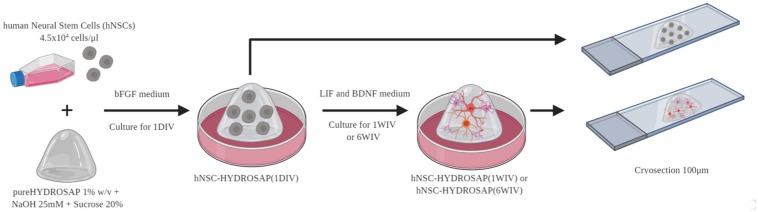
Experimental design of hNSC-HYDROSAP samples. Schematic representation of protocol used to generate hNSC-HYDROSAP. pureHYDROSAP (1%w/v) supplemented with NaOH and sucrose was mixed with hNSCs (4,5 × 10^4^ cells/μl). A droplet of 40 μl was placed in 24-wells with serum-free medium supplemented with bFGF and, after 2 days, shifted to a medium with LIF and BDNF supplements. hNSC-HYDROSAP was maintained in culture for 1DIV (1 day *in vitro*), 1WIV (1 week *in vitro*) and 6WIV (6 weeks *in vitro*) generating respectively hNSC-HYDROSAP(1DIV), hNSC-HYDROSAP(1WIV) and hNSC-HYDROSAP(6WIV). Finally, samples were cryosectioned at 100 μm for the subsequent immunostaining.

Human neural stem cells used in this work derived from four different human fetal brain tissue specimens. hNSCs were separately cultured at density of 10^4^ cells/cm^2^ using serum-free medium in the presence of basic fibroblast growth factor (bFGF; PeproTech) and epidermal growth factor (EGF; PeproTech) at final concentrations of 10 and 20 ng/ml, respectively. Cell cultures were maintained at 37°C, 5% O_2_ and 5% CO_2_ to support hNSC proliferation. Neurospheres were mechanically dissociated every 10–15 days in the same growth medium. hNSC-HYDROSAP samples were prepared as previously described (Marchini et al., 2019). Briefly, 0.81 mM pureHYDROSAP (dissolved 1% w/v in distilled water, GIBCO) was mixed with sucrose, NaOH and culture medium containing a cell density of 4.5 × 10^4^ cells/μl. A droplet of 40 μl was placed in a 24-well and serum-free medium supplemented with bFGF (20 ng/ml, PeproTech) was added to generate 3D scaffold. At 2 days *in vitro*, medium was shifted with a basal medium supplemented with leukemia inhibitory factor (LIF; 20 ng/ml, Chemicon) and brain derived neurotrophic factor (BDNF; 20 ng/ml, PeproTech). Fresh medium was replaced every 3 days. See Figure 1 for details.

### Preparation of 2D Culture

Human neural stem cells lines were seeded on the surface of Cultrex-BME^®^ (R&D Systems) at 3 × 10^4^ cells/cm^2^ and cultured for 1WIV. hNSCs were differentiated with basal medium (without growth factors) supplemented with bFGF (10 ng/ml). At 2DIV, bFGF medium was replaced with basal medium supplemented with LIF (20 ng/ml) and BDNF (20 ng/ml). Immunofluorescence analysis were performed as described for hNSC-HYDROSAP in the following section.

### Immunocytochemistry and Quantification

Human neural stem cells-HYDROSAP samples were fixed at 1DIV, 1WIV, and 6WIV with 4% paraformaldehyde, embedded in OCT and cryosectioned at 100 μm. For immunofluorescence analyses, sections were permeabilized with 0.3% Triton X-100 for 10 min at 4°C and blocked with 10% normal goat serum (GIBCO) for 1 h at room temperature. The following primary and secondary antibodies were used: mouse antiβIII-Tubulin (1:500, BioLegend), rabbit antiβIII-Tubulin (1:500, Covance) rabbit anti-glial fibrillary acidic protein (GFAP) (1:500, DAKO), mouse anti-galactocerebroside (GalC) (1:200, Millipore), mouse anti-oligodendrocytes marker O4 (1:200, Millipore), rabbit anti-Nestin (1:500, Millipore), mouse anti-microtubule associated protein 2 (MAP2) (1:300, Invitrogen), rabbit anti-growth associated protein-43 (GAP43) (1:100, Millipore), mouse anti-neurofilament-H (SMI31) (1:1000, BioLegend), rabbit anti-g-aminobutyric acid (GABA) (1:500, Sigma-Aldrich), rabbit anti-vescicular glutamate transporter 1 (VGLUT1) (1:500, Invitrogen), rabbit anti-choline acetyltransferase (ChAT, Invitrogen) (1:500), mouse anti-MBP (1:300, BioLegend), and rabbit anti-Ki67 (1:750, Invitrogen). To reveal primary antibodies, the following secondary antibodies were used: goat anti-rabbit Cy3 (1:1000, Jackson), goat anti-mouse Cy3 (1:1000, Jackson), goat anti-rabbit Alexa 488 (1:1000, Invitrogen), and goat anti-mouse Alexa 488 (1:1000, Invitrogen). The protocol for TUNEL assay (*in situ* cell death detection kit fluorescein, Roche) was performed following manufacturer’s instructions. Briefly, sections were permeabilized and incubated with TUNEL reaction mixture (1:10 in enzyme solution) for 1 h at 37°C.

A minimum of three fields for three independent experiments were chosen randomly. The acquisitions were performed at 40× magnification via Zeiss Microscope with Apotome System. Quantitative analyses were performed by counting manually positive cells for each marker using NIH-ImageJ software.

### Statistical Analysis

All data are represented as means ± SEM. Data sets were analyzed using GraphPad Prism 7 software: two-way ANOVA followed by a Tukey post-test were used for comparisons between the different groups; two-way ANOVA followed by Bonferroni post-test were used for comparisons between 2D-cultured hNSC on Cultrex and 3D-cultured hNSCs in HYDROSAP. *p*-value < 0.05 was considered as statistically significant.

## Results

### Differentiation of hNSC Lines in 3D Scaffolds of Multi-Functionalized Self-Assembling Peptides

In this work, four brain-derived human GMP-grade NSC lines (named hNSC#1, #2, #3, and #4) were compared to evaluate cell survival, neural differentiation and progressive neuronal maturation in serum-free conditions when cultured in functionalized 3D-microenvironment made of SAPs. hNSC-HYDROSAP samples were produced as previously descripted (Marchini et al., 2019) to obtain long-term cultures of densely seeded hNSCs. A schematic representation of experimental protocol is portrayed in Figure 1 and largely described in section “Materials and Methods.” hNSCs at high concentration (4.5 × 10^4^ cells/μl) were encapsulated in pureHYDROSAP, composed by multifunctionalized SAPs and branched SAPs, supplemented with sucrose and sodium hydroxide (NaOH) solutions. Three representative timepoints were tested: 1DIV, 1WIV, and 6WIV. Serum-free long-term cultures were cryosectioned and subsequently characterized via immunofluorescence analyses. To deeply characterize the difference among four independent hNSC lines *in vitro*, results were divided in two parts: (1) comparisons among hNSC lines at each timepoint (1DIV, 1WIV and 6WIV); (2) time-tracking of marker expression for each hNSC line.

**FIGURE 2:**
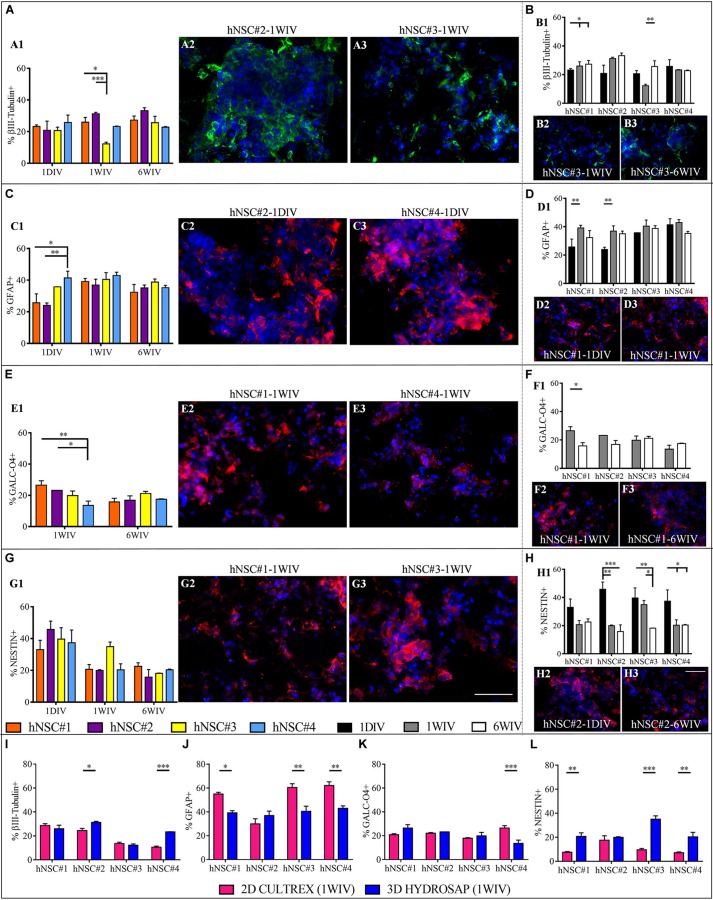
Neural differentiation of hNSC-HYDROSAP samples in 3D culture system and comparison with 2D CULTREX. Quantification of positive cells for βIII-Tubulin (neurons) **(A)**, GFAP (astrocytes) **(C)**, GALC-O4 (oligodendrocytes) **(E)** and Nestin (stem/progenitor cells) **(G)** relating to the analyzed hNSC lines. The graphs on the left **(A1,C1,E1,G1)** highlight the differences between the four hNSC lines at three timepoints [1DIV (1 day *in vitro*), 1WIV (1 week *in vitro*) and 6WIV (6 weeks *in vitro*)] showing a similar expression of βIII-Tubulin, GFAP and GALC-O4 markers at 6WIV for all cell lines. Histograms on the right **(B1,D1,F1,H1)** show time-tracking of percentage of cells positive for the abovementioned markers and reveal a trend of decreasing progenitor cell population over time for each cell lines. **(I–L)** Comparison between 2D cell culture with cells seeded on CULTREX and 3D cell cultures of hNSCs encapsulated in HYDROSAP at 1WIV. Percentages of cells positive for βIII-Tubulin **(I)**, GFAP **(J)**, GALC/O4 **(K)**, and Nestin **(L)** markers. After 1WIV in culture, results showed a lower expression of GFAP marker, a higher number of Nestin+ cells and an increased population of cells positive for βIII-Tubulin (for hNSC#2 and hNSC#4) in 3D cell cultures compared to 2D. All graphs show mean ± SEM of triplicate experiments. Statistical analysis shows significant differences (*p < 0.05; **p < 0.01; ***p < 0.001). Fluorescence images represent the main significant differences specified in the corresponding histogram: βIII-Tubulin in green **(A2,A3,B2,B3)**, GFAP in red **(C2,C3,D2,D3)**, GALC-O4 in red **(E2,E3,F2,F3)**, and NESTIN in red **(G2,G3,H2,H3)**. Cell nuclei are costained with DAPI (blue). (Scale bar, 50 μm).

Firstly, the capability of hNSCs to differentiate into neurons, astrocytes and oligodendrocytes and stemness maintenance were assessed over time (see Supplementary Figures 1, 2). Immature population of neurons was evaluated through βIII-Tubulin marker. Comparison between cell lines (Figure 2A and Supplementary Table S1) exhibited a percentage of βIII-Tubulin+ cells in hNSC#3 (12.2 ± 1.1%) significantly lower than in hNSC#2 (31.3 ± 0.87) and hNSC#1 (25.1 ± 2.97%) at 1WIV. Interesting results were obtained by time-tracking βIII-Tubulin expression: only hNSC#1 and hNSC#3 showed increasing expression over time (Figure 2B and Supplementary Table S1).

Expression of GFAP marker displayed significant differences at 1DIV, i.e., a higher percentage of +cells in hNSC#4 (41.37 ± 4.3%) compared to hNSC#2 (24.01 ± 1.5%), and hNSC#1 (25.67 ± 5.7%) (Figure 2C and Supplementary Table S1). Population of astrocytes significantly grew up for hNSC#1 and hNSC#2 over time from 1DIV to 1WIV in culture (Figure 2D and Supplementary Table S1).

GALC-O4+ cells were not present at 1DIV, as previously demonstrated (Marchini et al., 2019), but they showed up at 1 week, when hNSC#1 and hNSC#2 exhibited greater percentage values than hNSC#4 (Figure 2E). The expression of oligodendrocytes markers decreased only in hNSC#1 (1WIV: 26.53 ± 2.8%; 6WIV: 15.72 ± 2.3%) (Figure 2F and Supplementary Table S1).

Human neural stem cells from all lines expressed similar (among the lines) Nestin+ cells at the tested three timepoints (∼38.9% at 1DIV, ∼31.9% at 1WIV, and ∼19.21% at 6WIV) (Figure 2G). Instead, there was a progressive decrease over time for all hNSC lines, with significant differences (over time) for hNSC#2, hNSC#3, and hNSC#4 (Figure 2H and Supplementary Table S1).

To understand if HYDROSAP can coax the fate of the analyzed hNSC lines and, for example, foster cell differentiation, a comparison between 2D (cells cultured on CULTREX) and 3D condition (cells encapsulated in HYDROSAP) was performed at 1 WIV (Figures 2I–L and Supplementary Table S2). hNSC#2 and hNSC#4 obtained a significant larger number of βIII-Tubulin+ cells in 3D compared to 2D condition (hNSC#2: 24.47 ± 1.48% for 2D and 31.28 ± 0.86% for 3D; hNSC#4: 10.58 ± 0.86% for 2D, and 23.28 ± 0.24% for 3D). Moreover, hNSC#1, hNSC#3, and hNSC#4 showed a significantly lower astrocytes population and larger Nestin+ cells in 3D preparation compared to 2D culture. Finally, expression of GALC/O4 marker was constant in 2D and 3D preparation with a value around 20% (except for hNSC#4).

### Neuronal Maturation Into GABAergic, Glutamatergic and Cholinergic Phenotypes at Different Timepoints *in vitro*

**FIGURE 3:**
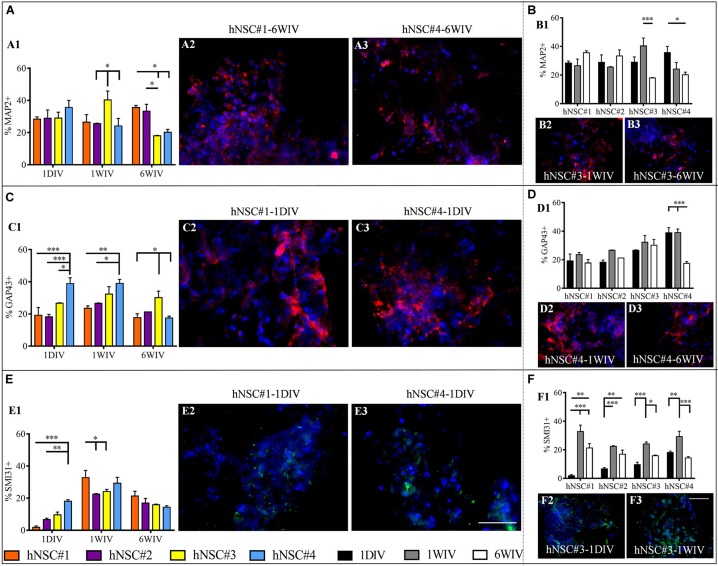
Quantitative evaluation of neuronal maturation. Data are expressed as percentages of cells positive for MAP2 (late neuronal marker) **(A1,B1)**, GAP43 (axonal growth cone marker) **(C1,D1)** and SMI31 (phosphorylated neurofilaments) **(E1,F1)**. Evaluation of markers expression showed differences among the hNSC lines **(A,C,E)**. Instead, Time-tracking of hNSC lines at 1DIV (1 day *in vitro*), 1WIV (1 week *in vitro*) and 6WIV (6 weeks *in vitro*) **(B,D,F)** revealed a stabilized expression of MAP2 **(B1)** and GAP43 **(D1)** markers over time for all hNSC lines except for hNSC#3 and hNSC#4, respectively [as shown in corresponding fluorescence images **(B2,B3,D2,D3)**], while SMI31 expression peaked at 1WIV in all cell lines **(F1)**. Representative fluorescence images display the most significant differences for MAP2 (red, **A2,A3,B2,B3**), GAP43 (red, **C2,C3,D2,D3**), and SMI31 (green, **E2,E3,F2,F3**) markers. Data are represented as mean ± SEM, *p < 0.05, **p < 0.01, ***p < 0.001. Scale bar, 50 μm.

Neuronal maturation of hNSCs cultured in HYDROSAP was verified by analyzing the expression of neuronal markers MAP2, GAP43, and SMI31 and by assessing the presence of inhibitory, excitatory and cholinergic neurons (GABA+, VGLUT1+, and ChAT+ cells, respectively) (see Supplementary Figures 3, 4, 5A). Neuron specific cytoskeletal protein MAP2 is expressed since the first day in culture in all hNSC lines. When hNSC-HYDROSAP samples were maintained in culture for 1WIV, hNSC#3 (40.33 ± 5.5%), reached a percentage significantly higher compared to hNSC#2 and hNSC#4 (25.5 ± 3.8% and 24.12 ± 4.64%, respectively). Finally, MAP2+ cells at 6WIV in hNSC#3 (17.95 ± 0.29%) and hNSC#4 (20.15 ± 1.88%) are lower than hNSC#1 (35.57 ± 1.38%) and hNSC#2 (33.32 ± 4.24%) (Figure 3A and Supplementary Table S1). Consequently, in these last cell lines MAP2 marker expression significantly decreased from 1WIV to 6WIV for hNSC#3 and from 1DIV to 6WIV for hNSC#4. On the other hand, hNSC#1 and hNSC#2 showed unchanged percentages of MAP2+ cells throughout the experimental timeframe (Figure 3B and Supplementary Table S1).

Moreover, hNSCs differentiated progeny showed expression of GAP43 (Figure 3C and Supplementary Table S1), a growth cone protein highly express during axon elongation: at 1DIV and 1WIV, hNSC#4 had a larger number of GAP43+ cells compared to other cell lines. Specifically, at 1DIV GAP43+ cells are significantly higher in hNSC#4 (38.85 ± 3.64%) compared to hNSC#3 (26.69 ± 0.25%, ^∗^*p* < 0.05), hNSC#2 (18.22 ± 1.55%), and hNSC#1 (19.21 ± 4.81%). On the other hand, in the case of hNSC#4, a significant decrease at 6WIV was detected compared to 1DIV and 1WIV (Figure 3D and Supplementary Table S1).

Neuronal maturation was confirmed by the expression of SMI31 phosphorylated neurofilament H. SMI31+ cells were detected at low percentage at 1 DIV. Similarly, to GAP43, SMI31 expression in hNSC#4 reached significantly higher values (18.09 ± 0.99%) than hNSC#2 and hNSC#1 (6.67 ± 0.87% and 1.8 ± 0.87%, respectively). However, SMI31+ cell percentages peaked in all hNSC lines at 1WIV, with values in hNSC#1 (32.84 ± 4.45%) significantly higher than in hNSC#2 and hNSC#3 (21.31 ± 3.01% and 15.89 ± 0.41, respectively) (Figures 3E,F and Supplementary Table S1).

**FIGURE 4:**
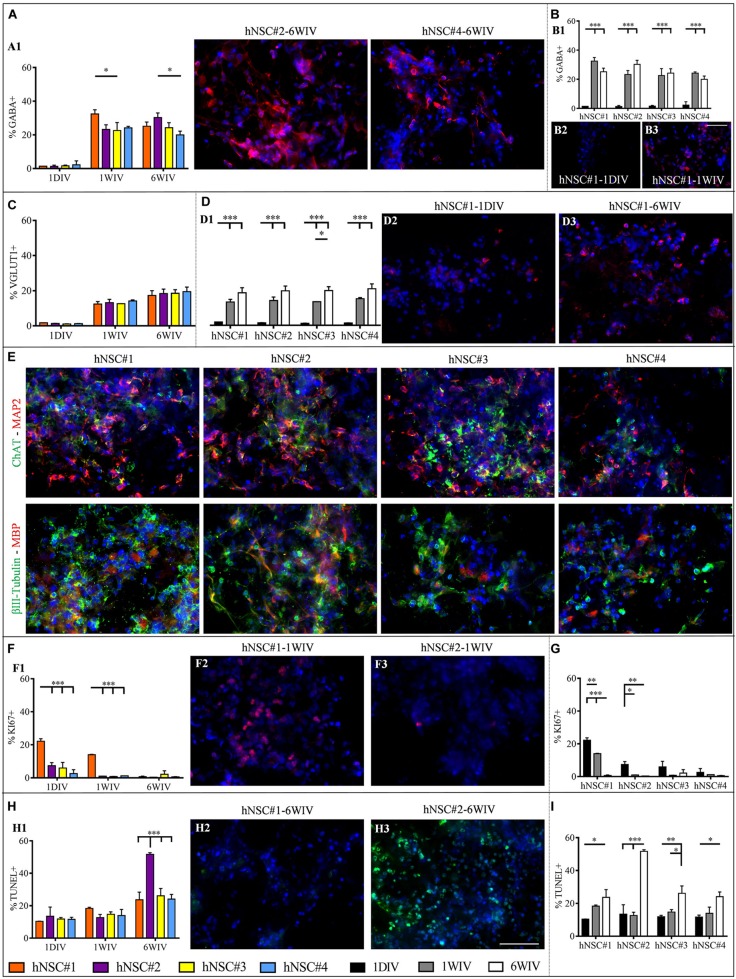
Analysis of neuronal subtypes, proliferation rate and percentage of apoptotic cells in hNSC#1, hNSC#2, hNSC#3, and hNSC#4 lines. Quantitative comparison of GABA+ cells among hNSC lines **(A)** and time-course study at 1DIV (1 day *in vitro*), 1WIV (1 week *in vitro*), and 6WIV (6 weeks *in vitro*) revealed a significant initial increment of GABA expression at 1WIV, subsequently plateauing till 6WIV **(B)**. **(C)** Evaluation of glutamatergic neurons showed no significant differences among the four cell lines. **(D)** Time-course study displays a progressively increased expression of VGLUT1 marker. **(E)** Confocal microscope images for ChAT+ (in green) and MAP2+ (in red) neurons and for βIII-Tubulin+ (in green) and MBP+ (in red) cells. Markers expression was verified for all hNSC lines cultured at 6WIV; fluorescence images display the typical morphologies of differentiated cells, the formation of an entangled neuronal networks and close expression of both neuronal and myelin markers. **(F)** Percentage of KI67+ cells over total number of cells for the four cells lines at 1DIV, 1WIV and, 6WIV. **(G)** All hNSC lines showed a progressive extinction of their proliferative status over time. **(H)** Cells viability test (TUNEL assay) for the hNSC lines at 1DIV, 1WIV, and 6WIV, featured a significantly higher percentage of TUNEL+ cells at 6WIV for hNSC#2. **(I)** Over time each hNSC line showed a progressive increase of apoptotic cells. Fluorescence images were chosen to highlight the main significant differences arising from the corresponding graph: GABA **(A2,A3,B2,B3)** VGLUT1 **(D2,D3)**, and KI67 **(F2,F3)** are marked in red, apoptotic cells **(H2,H3)** in green and cell nuclei (DAPI) in blue colors. Data represented as mean ± SEM, *p < 0.05, **p < 0.01, ***p < 0.001. Scale bar, 50 μm.

*In vitro* maturation tests revealed a highly abundant population of GABAergic, glutamatergic and cholinergic neurons. GABA+ cells were expressed since day 1, albeit in a very low percentage (∼1.5%), in all cell lines. At 1WIV and 6WIV the four hNSC-HYDROSAP samples showed similar high percentages of GABAergic neurons, with few significant differences among the four lines (Figure 4A). In all lines the presence of GABA+ cells increased over time from 1DIV to 1WIV and 6WIV (Figure 4B and Supplementary Table S1). The four cell lines displayed equable percentages of VGLUT1+ cells at all analyzed timepoints (Figure 4C), with a significant increase over time (Figure 4D and Supplementary Table S1) and an average percentage of glutamatergic neurons of 19% at 6WIV. Choline acetyltransferase (ChAT) positive neurons were detected in all cell lines at 6WIV and colocalization with MAP2+ neurons are represented in Figure 4E. Intriguingly, expression of MBP, a major myelin constituent produced by mature oligodendrocytes, was found in conjunction with βIII-Tubulin positive neurons showing entangled networks of positive cells as pictured in Figure 4E.

### Proliferation Rate and Cell Survival in All hNSC Lineages

Viability (TUNEL Assay) and proliferative state (KI67 marker) of hNSC lines were evaluated at 1DIV, 1WIV and 6WIV by immunostaining (Supplementary Figures 5B, 6). Percentages of proliferating cells (KI67+ cells) were modest in all cell lines at all timepoints, except for hNSC#1 at 1DIV and 1WIV (22.19 ± 1.49% and 14.02 ± 0.22%, respectively) (Figure 4F). As expected, a downregulation of proliferative marker occurred over time due to the ongoing differentiation and maturation of the seeded hNSCs. This evidence was clear in hNSC#1, hNSC#2, and hNSC#4 (Figure 4G and Supplementary Table S1).

TUNEL assay, targeting cell viability inside 3D HYDROSAP scaffolds showed low values in all cell lines. However, hNSC#2 at 6WIV reached a percentage of apoptotic cells (51.67 ± 1.01%) significantly higher than in hNSC#1 (23.69 ± 4.67%), hNSC#3 (26.11 ± 4.51%), and hNSC#4 (24.08 ± 2.91%) (Figure 4H and Supplementary Table S1). This shared trend of increasing TUNEL+ cells over time is clearly highlighted by Figure 4I for all hNSC lines.

## Discussion

We tackled the issue of reproducibility of data we previously published with our unique 3D hNSC culture system, obtained with a single hNSC line. Here, we extended the analysis to other hNSC lines, all derived from fetal brain tissue from distinct fetuses, produced according to GMP standards and candidate for usage in clinical trials for the treatment of neurological disorders (Mazzini et al., 2015, 2019; Vescovi, 2017). Biophysical and mechanical support for hNSCs was provided by pureHYDROSAP, a synthetic ECM-like scaffold, entirely made of biomimetic SAPs, with mechanical properties in the range of nervous tissue (100–1000 Pa) (Arani et al., 2015). A lot of works uses animal-derived scaffolds (like Matrigel or Cultrex) as embedding matrices for 3D constructs for their remarkable capability to affect cell behavior enabling excellent cell adhesion and spreading. Nevertheless, they have a limited reproducibility due to the lot-to-lot variability, lack of well-defined composition and minimal tailorability for different tissue types (Knight and Przyborski, 2015). Conversely, SAPs parameters (AA sequences, concentration, pH, and so forth) can be easily controlled allowing the design of peptide hydrogel systems with tailor-made properties (Pugliese and Gelain, 2017). Indeed, we used a synthetic matrix composed of (1) linear standard (LDLK)_3_ SAP, (2) two pro-regenerative functionalized linear SAPs (Ac-KLP-(LDLK)_3_ (Caprini et al., 2013) and Ac-SSL-(LDLK)_3_ (Gelain et al., 2012), and (3) three-branched (LDLK)_3_ SAPs (Pugliese et al., 2018a). By adding sucrose to increase osmotic pressure and NaOH to reach physiological pH, the multifunctionalized mixture was proved to be an optimal support for differentiation and maturation of a hNSC line, forming the already defined hNSC-HYDROSAP.

Here we tested additional four hNSC lines at three timepoints in 3D culture setups: (1) 1DIV, i.e., early-stage differentiation when cells are depleted of EGF; (2) 1WIV, standard time needed for hNSCs, exposed to LIF and BDNF supplements in the culture media, to create a relevant neuronal network: (3) 6WIV, proven to be the optimal time *in vitro* in terms of hNSCs progeny maturation and formation of an entangled electrically active neuronal network (Marchini et al., 2019). Results were grouped by comparing the different hNSC lines at each time point and by time-tracking each hNSC differentiation over time.

Characterization of cell viability, proliferation, differentiation, and maturation showed various significant differences. First, we verified the multipotency of hNSC lines encapsulated in 3D HYDROSAP, characterizing the presence of βIII-Tubulin positive neurons, GFAP positive astrocytes and GALC/O4 positive oligodendrocytes at 1DIV, 1WIV, and 6WIV (Figure 2). Interestingly, hNSC#1 showed a progressive increase over time of neuron and astrocytes, with concomitant decreased of GALC/O4+ cells. Moreover, Nestin positive cells decreased over time for all cell lines except for hNSC#1: this is likely ascribable to an initially higher proliferation rate (until 1WIV) caused by an enriched percentage of stem/progenitor cells population that progressively faded into differentiating (or fate-committed) cells (Figure 4G). To note, the obtained values can be easily modified with further customizations of HYDROSAP depending on the regenerative application: indeed we already demonstrated that by tuning different key experimental parameters (e.g., matrix stiffness, presence of functional motifs, and/or addition of neurotrophic factors) it is possible to modulate NSCs differentiation and improve differentiation toward neuronal fate (Jain et al., 2020): for example, we showed that increments of cell proliferation rate and neuronal differentiation is inversely proportional to the matrix stiffness, while stiffness and astrocytes number directly correlated (Cunha et al., 2011; Caprini et al., 2013; Han et al., 2020; Pandamooz et al., 2020). In future experiments, it will also be interesting to assess the influence of time-evolving viscoelastic properties of hydrogels over cellular behavior (Mattei et al., 2020).

Despite 2D culturing systems may give unrealistic information on cell morphology and cellular functions *in vivo*, they can still be useful for cell-based studies. Indeed, 2D cultures on CULTREX are routinely used to evaluate cell differentiation and/or proliferation. Besides, by analyzing previous data on 3D CULTREX (Marchini et al., 2019) with current results on 2D CULTREX we detected a higher percentage of Nestin+ cells and a negative trend for GFAP markers when hNSCs were cultured in 3D environment. In the same way, if compared to 2D cultures on coatings of CULTREX at 1WIV (Figures 2I–L), our 3D culture system, made of multi-functionalized SAPs, coaxed an enhancement in neurons (for hNSC#2 and hNSC#4), a reduction of astrocytic phenotype and increased percentages of Nestin+ cells (except for hNSC#2), all desired effects for a neuroregenerative therapy aimed at transplanting neuronal, oligodendroglial or cell progenitor cells (Raspa et al., 2016; Zheng et al., 2020). Therefore, in line with previous publications, “soft” 3D culture systems foster the maintenance of stem cells *in vitro* (Mari-Buye and Semino, 2011; Sahab Negah et al., 2017).

A dense network of long neuronal branched processes was strengthened by late-stage neuronal marker MAP2, expressed since day 1 post-induction of differentiation (Ilieva et al., 2013): also, at 6WIV MAP2+ cell population in hNSC#1 and hNSC#2 was larger than in hNSC#3 and hNSC#4.

These results were corroborated by the staining against GAP43, a marker expressed in growing axonal terminal (Morita and Miyata, 2013), where GAP43+ cells decreased at 6WIV, likely suggesting an on-going process of maturation of neurons differentiated from hNSCs (Figure 3D). Phosphorylated neurofilaments were present in all cell lines, peaking at 1WIV and decreasing at 6WIV to approximately 20% for all hNSC lines (Figures 3E,F).

Most interesting results were obtained on the expression of neuronal phenotypes at 6WIV: all hNSC lines showed a fraction of glutamatergic cells (∼20% at 6WIV), a fraction of GABAergic cells (∼25% at 6WIV) and a fraction of cholinergic cells. Specifically, percentage of GABA and VGLUT1 markers gradually increased over time for all cell lines, thus indicating a progressive neuronal maturation (Figures 4B,D). Additionally, the presence of MBP+ cells surrounding protruding axons testified a good level of maturation, and maybe an initial phase of myelination *in vitro*, of hNSC seeded in HYDROSAP synthetic scaffold in serum-free conditions (Figure 4E).

Despite some differences due to their genetic background (Kim et al., 2016), all cell lines have responded with a remarkable cell proliferation, an expected decrease in stemness overtime, a robust expression of MAP2, GAP43, and SMI31 neuronal markers, together with an important expression of GABAergic, glutamatergic and cholinergic neurons and mature MBP+ oligodendrocytes, all within a successfully standardized and reproducible entangled and randomly organized 3D neural network.

However, some morphological differences of neuronal phenotypes were detected at 6WIV and depicted in Figure 4E. Qualitative investigation with MAP2 marker for mature neurons and immature neuronal marker βIII-Tubulin revealed extended axonal processes for hNSC#1, hNSC#2, and hNSC#3; while hNSC#4 progeny showed poorly branched neurons instead.

Also, a possible refinement of the current technique to overcome the progressive increment of cell apoptosis, could be the usage of microfluidic devices (Mofazzal Jahromi et al., 2019) or spinner flasks (Newland et al., 2020) to increase oxygen and nutrients exchange inside the 3D culture system. In turn, this could also ameliorate cell maturation yielding out more entangled, and likely electrically active neuronal networks to be confirmed by electrophysiology tests.

In addition, neurotrophins and growth factors loaded into SAPs hydrogel may further influence hNSCs commitment and differentiation into specific neural phenotypes and may generate multi-layer structure mimicking the cyto-organization of CNS tissue (Nierode et al., 2019). This construct may be used as an *in vitro* platform to investigate tissue morphogenesis and neural development, but also for neuropharmacology studies and likely, if used for neural progenitors cultures from induced-pluripotent stem cells (Rosati et al., 2018) from a living donor, to diagnose neural pathologies in patients in a near future.

Lastly, given the satisfactory reproducibility of the obtained *in vitro* results, similar also to previously published data (Marchini et al., 2019), this construct can be potentially used in spinal cord injury models to ameliorate motor sensory recovery and reconstruct the synaptic connections of interrupted neural pathways.

## Data Availability Statement

The raw data supporting the conclusions of this article will be made available by the authors to any qualified researcher at reasonable request.

## Ethics Statement

This study involve the use of human tissue collected from spontaneous miscarriages; tissue procurement was approved by the Ethical Commitee of Umbria’ Health Institutes (CEAS) and with written consent of mother.

## Author Contributions

AM and FG designed the project and wrote the manuscript. AM and CF performed experiments and analyzed the data. FG supervised the project.

## Conflict of Interest

The authors declare that the research was conducted in the absence of any commercial or financial relationships that could be construed as a potential conflict of interest.

## Abbreviations

BDNF: brain derived neurotrophic factor
bFGF: basic fibroblast growth factor
CNS: central nervous system
DIV: days *in vitro*
ECM: extracellular matrix
EGF: epidermal growth factor
GMP: good manufacturing practice
hNSCs: human neural stem cells
HYDROSAP: multifunctionalized self-assembling peptide with sucrose and sodium hydroxide solution
LIF: leukemia inhibitory factor
pureHYDROSAP: multifunctionalized self-assembling peptide
SAP: self-assembling peptide
3D: three-dimensional
2D: two-dimensional
WIV: weeks *in vitro*.
