# Supplementary material for: Multi-Functionalized Self-Assembling Peptides as Reproducible 3D Cell Culture Systems Enabling Differentiation and Survival of Various Human Neural Stem Cell Lines
Source: Front Neurosci. 2020 May 5;14:413. doi: 10.3389/fnins.2020.00413 (PMC7214803; doi:10.3389/fnins.2020.00413)
Supplement: Supplementary file 1 [file Data_Sheet_1.docx]

Supplementary Material

Supplementary Figures


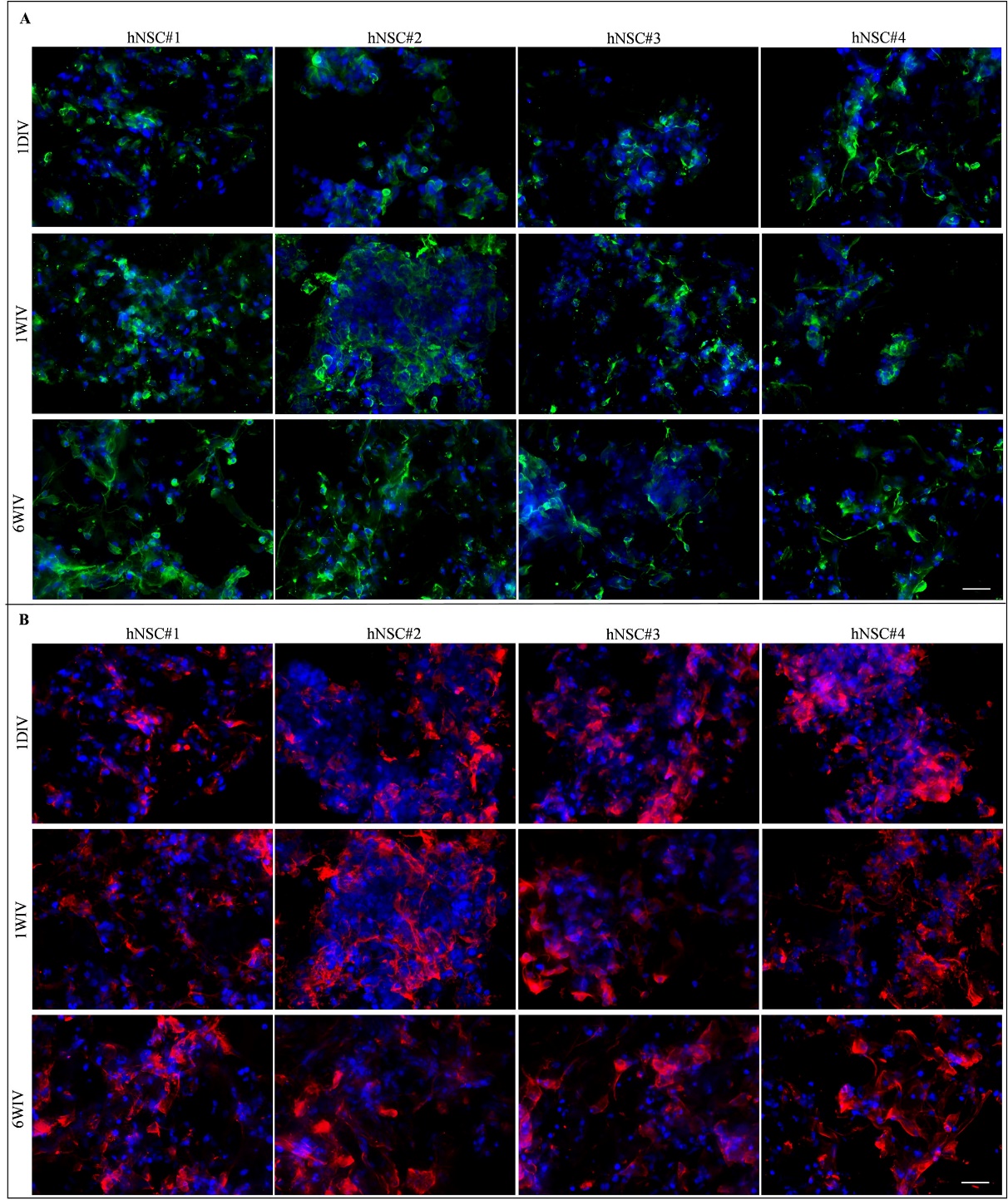


**Supplementary Figure 1.** Representative images of βIII-Tubulin (in green) (**A**) and GFAP (in red) (**B**) markers for hNSC#1, hNSC#2, hNSC#3 and hNSC#4 for each time point: 1DIV (day in vitro), 1WIV (weeks in vitro) and 6WIV (weeks in vitro). Cell nuclei are costained with DAPI (blue). Scale bar, 50 μm.


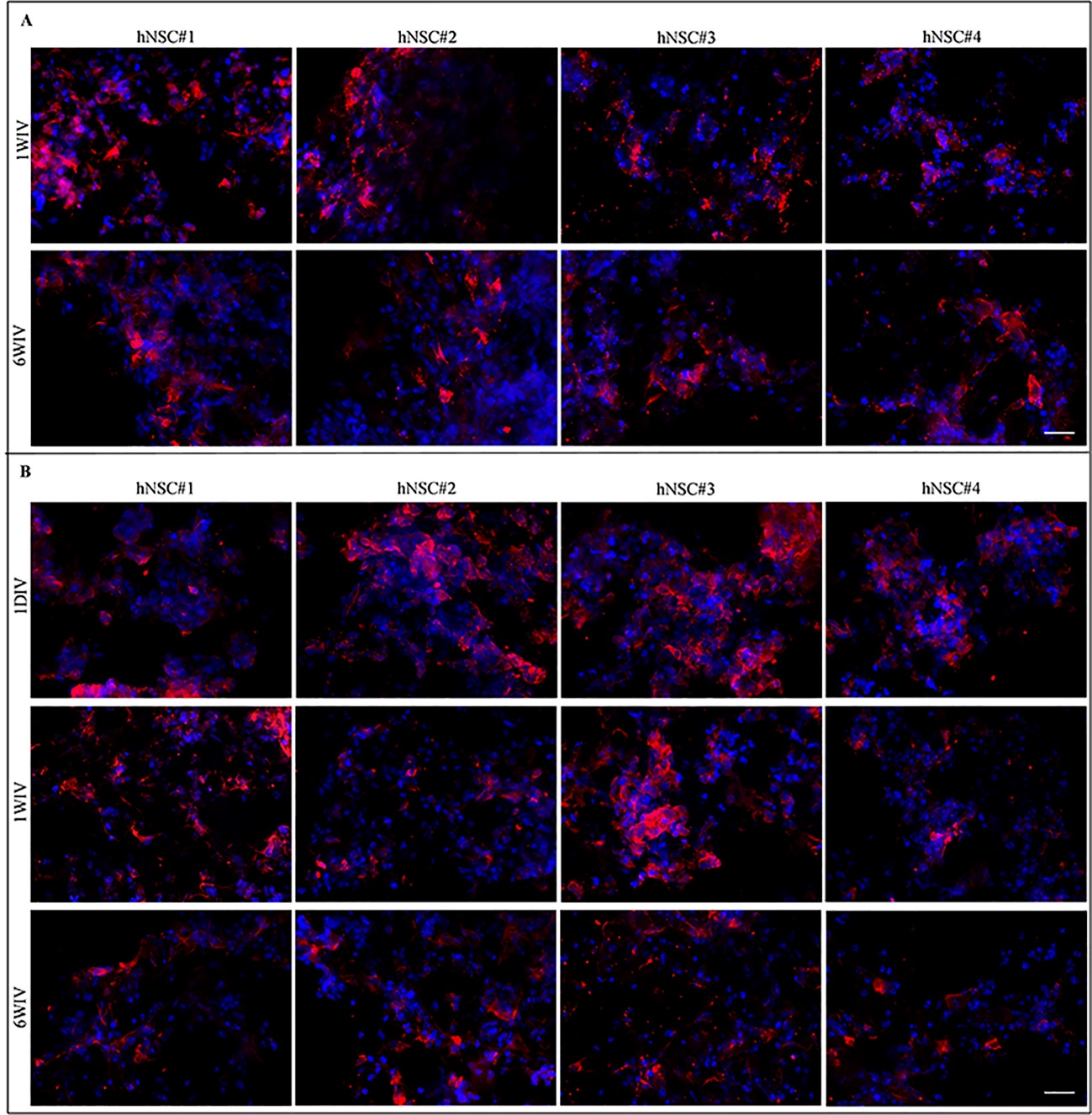


**Supplementary Figure 2.** Representative images of GALC/O4 (in red) (**A**) and NESTIN (in red) (**B**) markers for hNSC#1, hNSC#2, hNSC#3 and hNSC#4 for each time point: 1DIV (day in vitro), 1WIV (weeks in vitro) and 6WIV (weeks in vitro). GALC-O4+ cells were not present at 1DIV. Cell nuclei are costained with DAPI (blue). Scale bar, 50 μm.


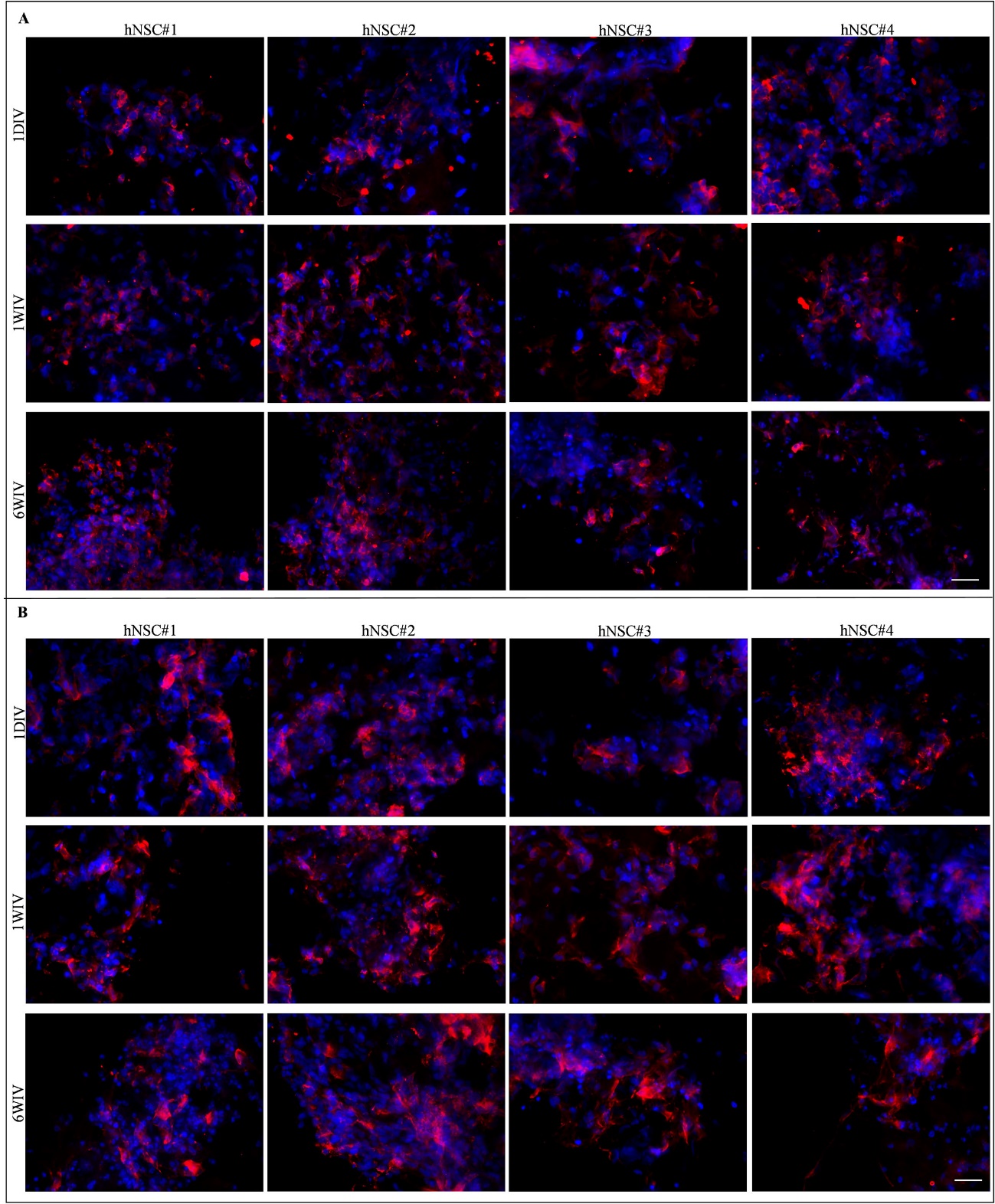


**Supplementary Figure 3.** Representative images of MAP2 (in red) (**A**) and GAP43 (in red) (**B**) markers for hNSC#1, hNSC#2, hNSC#3 and hNSC#4 for each time point: 1DIV (day in vitro), 1WIV (weeks in vitro) and 6WIV (weeks in vitro). Cell nuclei are costained with DAPI (blue). Scale bar, 50 μm.


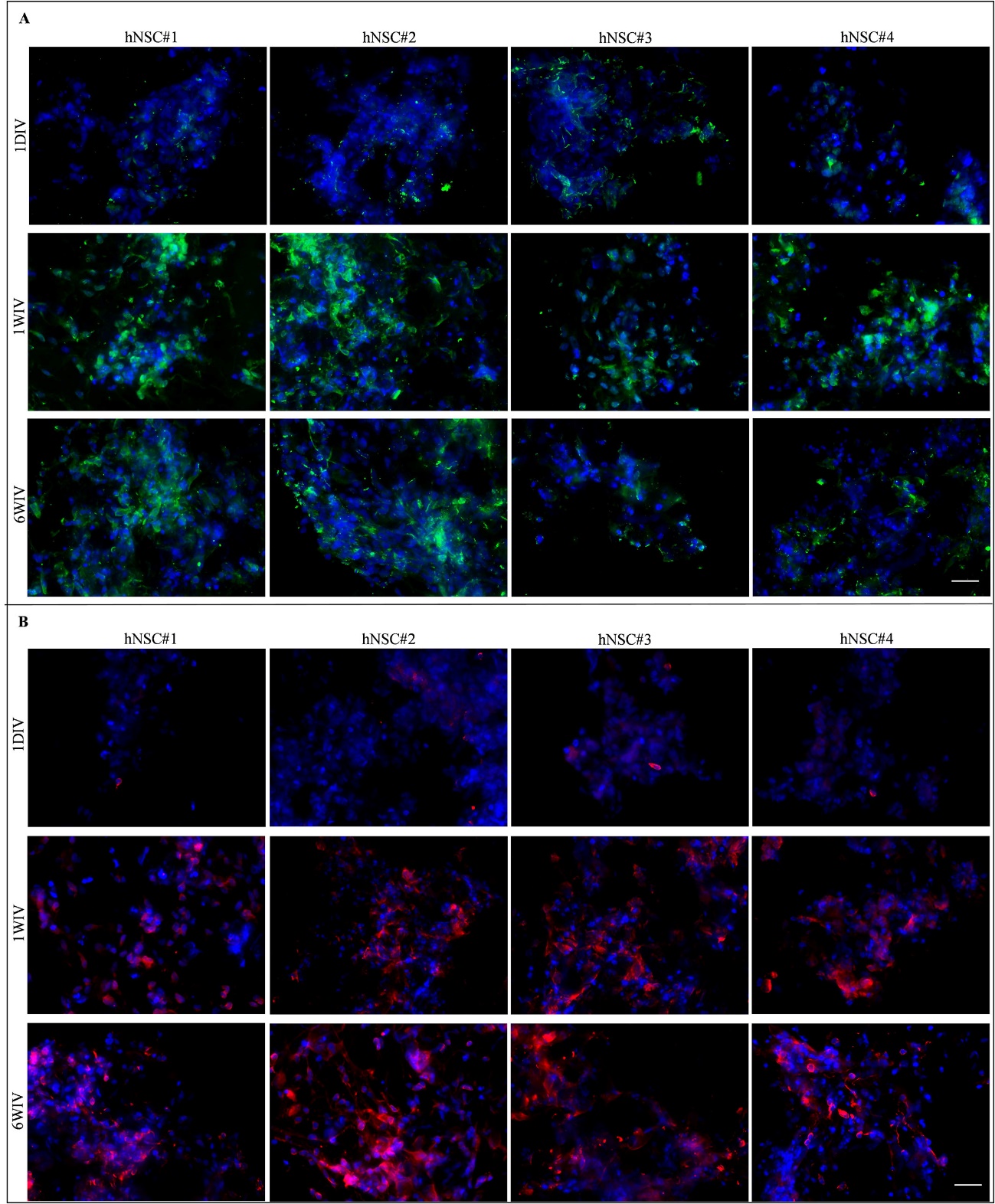


**Supplementary Figure 4.** Representative images of SMI31 (in green) (**A**) and GABA (in red) (**B**) markers for hNSC#1, hNSC#2, hNSC#3 and hNSC#4 for each time point: 1DIV (day in vitro), 1WIV (weeks in vitro) and 6WIV (weeks in vitro). Cell nuclei are costained with DAPI (blue). Scale bar, 50 μm.


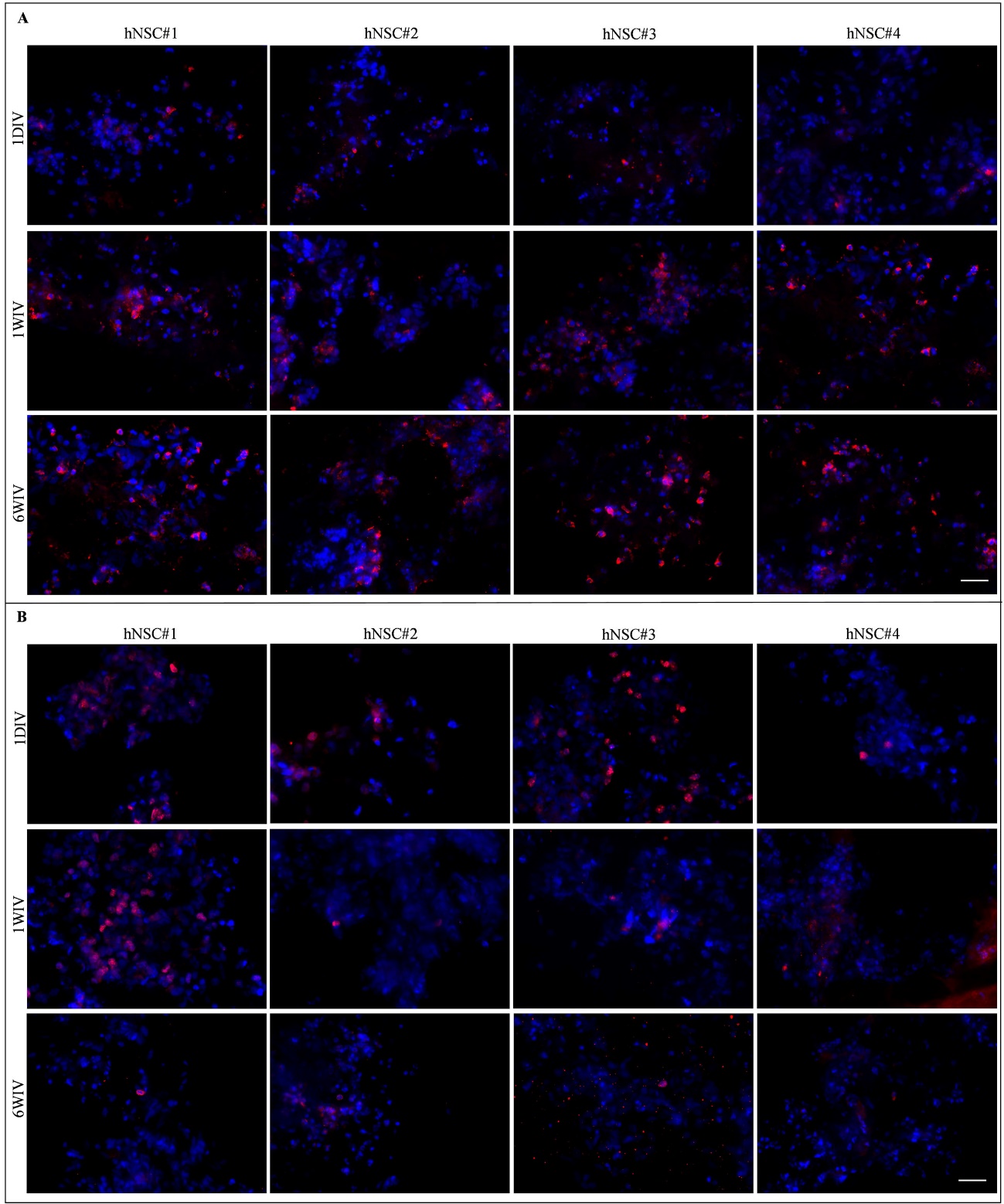


**Supplementary Figure 5.** Representative images of VGLUT1 (in red) (**A**) and KI67 (in red) (**B**) markers for hNSC#1, hNSC#2, hNSC#3 and hNSC#4 for each time point: 1DIV (day in vitro), 1WIV (weeks in vitro) and 6WIV (weeks in vitro). Cell nuclei are costained with DAPI (blue). Scale bar, 50 μm.


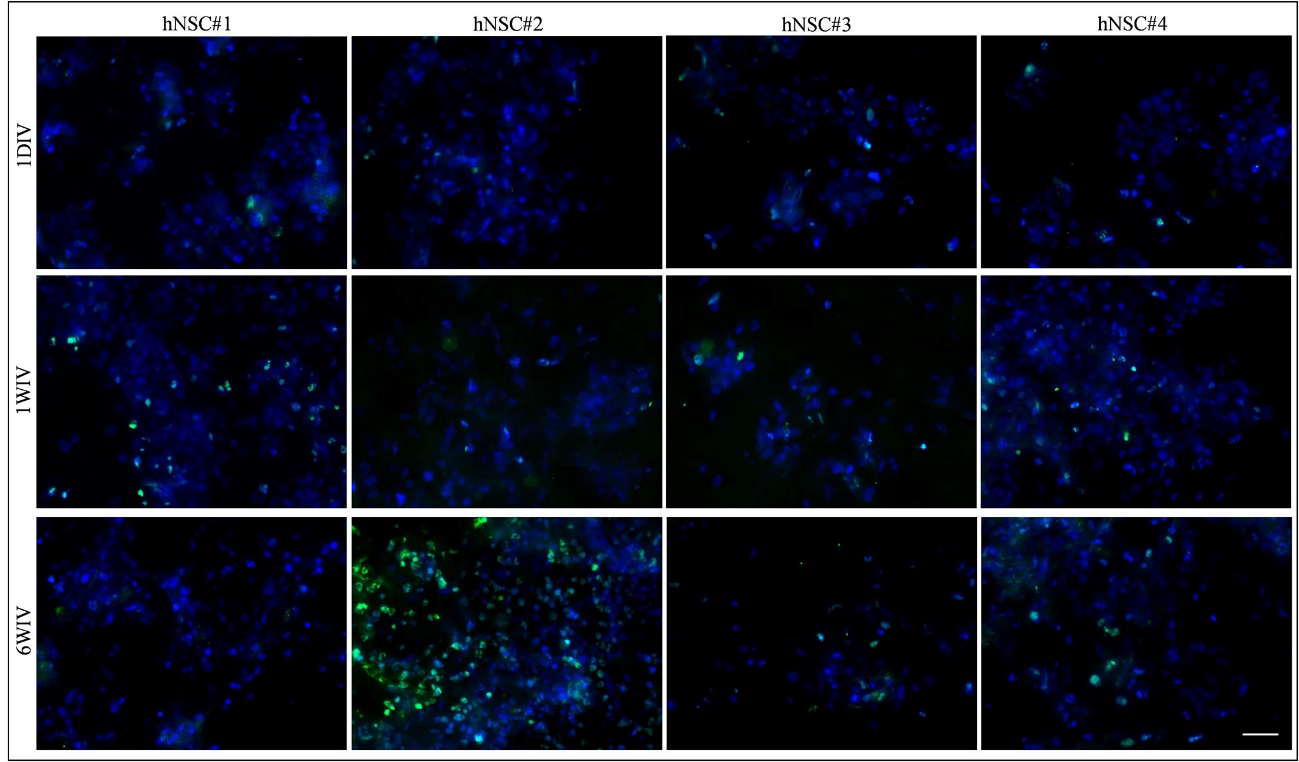


**Supplementary Figure 6.** Representative images of TUNEL ASSAY (in green) for hNSC#1, hNSC#2, hNSC#3 and hNSC#4 for each time point: 1DIV (day in vitro), 1WIV (weeks in vitro) and 6WIV (weeks in vitro). Cell nuclei are costained with DAPI (blue). Scale bar, 50 μm.

**Supplementary Table**

**TABLE S1** Detailed statistical analysis for all markers (two-way ANOVA followed by a Tukey post-test) by comparing the four hNSC line at three timepoints and by time-tracking each hNSC differentiation over time. Significant differences were expressed with YES; ‘ns’ stands for not statistically significant; p is p-value.

**βIII-Tubulin**

|  | 1DIV | 1WIV | 6WIV |
| --- | --- | --- | --- |
| hNSC#1 vs. hNSC#2 | ns (p=0.93) | ns (p=0.57) | ns (p=0.46) |
| hNSC#1 vs. hNSC#3 | ns (p=0.92) | YES (*p=0.01) | ns (p=0.98) |
| hNSC#1 vs. hNSC#4 | ns (p=0.92) | ns (p=0.91) | ns (p=0.69) |
| hNSC#2 vs. hNSC#3 | ns (p>0.99) | YES (***p<0.001) | ns (p=0.26) |
| hNSC#2 vs. hNSC#4 | ns (p=0.62) | ns (p=0.23) | ns (p=0.07) |
| hNSC#3 vs. hNSC#4 | ns (p=0.61) | ns (p=0.05) | ns (p=0.89) |

|  | hNSC#1 | hNSC#2 | hNSC#3 | hNSC#4 |
| --- | --- | --- | --- | --- |
| 1DIV vs. 1WIV | ns (p=0.77) | YES (*p=0.04) | ns (p=0.11) | ns (p=0.82) |
| 1DIV vs. 6WIV | ns (p=0.59) | YES (*p=0.01) | ns (p=0.45) | ns (p=0.75) |
| 1WIV vs. 6WIV | ns (p=0.95) | ns (p=0.88) | YES (**p=0.008) | ns (p>0.99) |

**GFAP**

|  | 1DIV | 1WIV | 6WIV |
| --- | --- | --- | --- |
| hNSC#1 vs. hNSC#2 | ns (p=0.98) | ns (p=0.96) | ns (p=0.94) |
| hNSC#1 vs. hNSC#3 | ns (p=0.15) | ns (p>0.99) | ns (p=0.51) |
| hNSC#1 vs. hNSC#4 | YES (*p=0.01) | ns (p=0.84) | ns (p=0.92) |
| hNSC#2 vs. hNSC#3 | ns (p=0.08) | ns (p=0.86) | ns (p=0.85) |
| hNSC#2 vs. hNSC#4 | YES (**p=0’005) | ns (p=0.56) | ns (p>0.99) |
| hNSC#3 vs. hNSC#4 | ns (p=0.62) | ns (p=0.95) | ns (p=0.86) |

|  | hNSC#1 | hNSC#2 | hNSC#3 | hNSC#4 |
| --- | --- | --- | --- | --- |
| 1DIV vs. 1WIV | YES (*p=0.02) | YES (*p=0.03) | ns (p=0.57) | ns (p=0.94) |
| 1DIV vs. 6WIV | ns (p=0.33) | ns (p=0.06) | ns (p=0.79) | ns (p=0.39) |
| 1WIV vs. 6WIV | ns (p=0.32) | ns (p=0.91) | ns (p=0.93) | ns (p=0.23) |

**GALC/O4**

|  | 1WIV | 6WIV |
| --- | --- | --- |
| hNSC#1 vs. hNSC#2 | ns (p=0.72) | ns (p=0.98) |
| hNSC#1 vs. hNSC#3 | ns (p=0.19) | ns (p=0.35) |
| hNSC#1 vs. hNSC#4 | YES (**p=0.004) | ns (p=0.95) |
| hNSC#2 vs. hNSC#3 | ns (p=0.72) | ns (p=0.55) |
| hNSC#2 vs. hNSC#4 | YES (*p=0.03) | ns (p>0.99) |
| hNSC#3 vs. hNSC#4 | ns (p=0.23) | ns (p=0.66) |

|  | hNSC#1 | hNSC#2 | hNSC#3 | hNSC#4 |
| --- | --- | --- | --- | --- |
| 1WIV vs. 6WIV | YES (*p=0.01) | ns (p=0.26) | ns (p>0.99) | ns (p=0.91) |

**NESTIN**

|  | 1DIV | 1WIV | 6WIV |
| --- | --- | --- | --- |
| hNSC#1 vs. hNSC#2 | ns (p=0.20) | ns (p>0.99) | ns (p=0.70) |
| hNSC#1 vs. hNSC#3 | ns (p=0.72) | ns (p=0.13) | ns (p=0.90) |
| hNSC#1 vs. hNSC#4 | ns (p=0.90) | ns (p>0.99) | ns (p=0.99) |
| hNSC#2 vs. hNSC#3 | ns (p=0.77) | ns (p=0.11) | ns (p=0.98) |
| hNSC#2 vs. hNSC#4 | ns (p=0.54) | ns (p>0.99) | ns (p=0.88) |
| hNSC#3 vs. hNSC#4 | ns (p=0.98) | ns (p=0.12) | ns (p=0.98) |

|  | hNSC#1 | hNSC#2 | hNSC#3 | hNSC#4 |
| --- | --- | --- | --- | --- |
| 1DIV vs. 1WIV | ns (p=0.14) | YES (**p=0.001) | ns (p=0.73) | YES (*p=0.03) |
| 1DIV vs. 6WIV | ns (p=0.24) | YES (***p<0.001) | YES (**p=0.006) | YES (*p=0.03) |
| 1WIV vs. 6WIV | ns (p=0.95) | ns (p=0.79) | YES (*p=0.03) | ns (p>0.99) |

**MAP2**

|  | 1DIV | 1WIV | 6WIV |
| --- | --- | --- | --- |
| hNSC#1 vs. hNSC#2 | ns (p>0.99) | ns (p>0.99) | ns (p=0.97) |
| hNSC#1 vs. hNSC#3 | ns (p>0.99) | ns (p=0.06) | YES (*p=0.01) |
| hNSC#1 vs. hNSC#4 | ns (p=0.51) | ns (p=0.97) | YES (*p=0.03) |
| hNSC#2 vs. hNSC#3 | ns (p>0.99) | YES (*p=0.04) | YES (*p=0.03) |
| hNSC#2 vs. hNSC#4 | ns (p=0.57) | ns (p>0.99) | ns (p=0.08) |
| hNSC#3 vs. hNSC#4 | ns (p=0.58) | YES (*p=0.02) | ns (p=0.97) |

|  | hNSC#1 | hNSC#2 | hNSC#3 | hNSC#4 |
| --- | --- | --- | --- | --- |
| 1DIV vs. 1WIV | ns (p=0.93) | ns (p=0.79) | ns (p=0.09) | ns (p=0.09) |
| 1DIV vs. 6WIV | ns (p=0.36) | ns (p=0.67) | ns (p=0.10) | YES (*p=0.02) |
| 1WIV vs. 6WIV | ns (p=0.20) | ns (p=0.30) | YES (***p<0.001) | ns (p=0.72) |

**GAP43**

|  | 1DIV | 1WIV | 6WIV |
| --- | --- | --- | --- |
| hNSC#1 vs. hNSC#2 | ns (p>0.99) | ns (p=0.86) | ns (p=0.81) |
| hNSC#1 vs. hNSC#3 | ns (p=0.25) | ns (p=0.14) | YES (*p=0.02) |
| hNSC#1 vs. hNSC#4 | YES (***p<0.001) | YES (**p=0.003) | ns (p>0.99) |
| hNSC#2 vs. hNSC#3 | ns (p=0.16) | ns (p=0.48) | ns (p=0.13) |
| hNSC#2 vs. hNSC#4 | YES (***p<0.001) | YES (**p=0.02) | ns (p=0.78) |
| hNSC#3 vs. hNSC#4 | YES (*p=0.02) | ns (p=0.35) | YES (*p=0.02) |

|  | hNSC#1 | hNSC#2 | hNSC#3 | hNSC#4 |
| --- | --- | --- | --- | --- |
| 1DIV vs. 1WIV | ns (p=0.53) | ns (p=0.10) | ns (p=0.34) | ns (p>0.99) |
| 1DIV vs. 6WIV | ns (p=0.92) | ns (p=0.73) | ns (p=0.65) | YES (***p<0.001) |
| 1WIV vs. 6WIV | ns (p=0.32) | ns (p=0.37) | ns (p=0.85) | YES (***p<0.001) |

**SMI31**

|  | 1DIV | 1WIV | 6WIV |
| --- | --- | --- | --- |
| hNSC#1 vs. hNSC#2 | ns (p=0.43) | YES (*p=0.01) | ns (p=0.51) |
| hNSC#1 vs. hNSC#3 | ns (p=0.09) | YES (*p=0.05) | ns (p=0.33) |
| hNSC#1 vs. hNSC#4 | YES (***p<0.001) | ns (p=0.67) | ns (p=0.15) |
| hNSC#2 vs. hNSC#3 | ns (p=0.79) | ns (p=0.95) | ns (p=0.99) |
| hNSC#2 vs. hNSC#4 | YES (**p=0.007) | ns (p=0.16) | ns (p=0.85) |
| hNSC#3 vs. hNSC#4 | ns (p=0.06) | ns (p=0.37) | ns (p=0.96) |

|  | hNSC#1 | hNSC#2 | hNSC#3 | hNSC#4 |
| --- | --- | --- | --- | --- |
| 1DIV vs. 1WIV | YES (***p<0.001) | YES (***p<0.001) | YES (***p<0.001) | YES (**p<0.004) |
| 1DIV vs. 6WIV | YES (***p<0.001) | YES  (**p<0.009) | ns (p=0.013) | ns (p=0.47) |
| 1WIV vs. 6WIV | YES  (**p=0.003) | ns (p=0.20) | YES  (*p=0.04) | YES (***p<0.001) |

**GABA**

|  | 1DIV | 1WIV | 6WIV |
| --- | --- | --- | --- |
| hNSC#1 vs. hNSC#2 | ns (p>0.99) | ns (p=0.06) | ns (p=0.46) |
| hNSC#1 vs. hNSC#3 | ns (p>0.99) | YES (*p=0.04) | ns (p>0.99) |
| hNSC#1 vs. hNSC#4 | ns (p>0.99) | ns (p=0.10) | ns (p=0.46) |
| hNSC#2 vs. hNSC#3 | ns (p>0.99) | ns (p>0.99) | ns (p=0.32) |
| hNSC#2 vs. hNSC#4 | ns (p>0.99) | ns (p>0.99) | YES (*p=0.03) |
| hNSC#3 vs. hNSC#4 | ns (p>0.99) | ns (p=0.97) | ns (p=0.62) |

|  | hNSC#1 | hNSC#2 | hNSC#3 | hNSC#4 |
| --- | --- | --- | --- | --- |
| 1DIV vs. 1WIV | YES (***p<0.001) | YES (***p<0.001) | YES (***p<0.001) | YES (***p<0.001) |
| 1DIV vs. 6WIV | YES (***p<0.001) | YES (***p<0.001) | YES (***p<0.001) | YES (***p<0.001) |
| 1WIV vs. 6WIV | ns (p=0.11) | ns (p=0.12) | ns (p=0.89) | ns (p=0.45) |

**VGLUT1**

|  | 1DIV | 1WIV | 6WIV |
| --- | --- | --- | --- |
| hNSC#1 vs. hNSC#2 | ns (p>0.99) | ns (p=0.99) | ns (p=0.97) |
| hNSC#1 vs. hNSC#3 | ns (p=0.99) | ns (p>0.99) | ns (p=0.95) |
| hNSC#1 vs. hNSC#4 | ns (p>0.99) | ns (p=0.87) | ns (p=0.78) |
| hNSC#2 vs. hNSC#3 | ns (p>0.99) | ns (p>0.99) | ns (p>0.99) |
| hNSC#2 vs. hNSC#4 | ns (p>0.99) | ns (p=0.97) | ns (p=0.96) |
| hNSC#3 vs. hNSC#4 | ns (p>0.99) | ns (p=0.89) | ns (p=0.97) |

|  | hNSC#1 | hNSC#2 | hNSC#3 | hNSC#4 |
| --- | --- | --- | --- | --- |
| 1DIV vs. 1WIV | YES (***p<0.001) | YES (***p<0.001) | YES (***p<0.001) | YES (***p<0.001) |
| 1DIV vs. 6WIV | YES (***p<0.001) | YES (***p<0.001) | YES (***p<0.001) | YES (***p<0.001) |
| 1WIV vs. 6WIV | ns (p=0.10) | ns (p=0.08) | YES (*p=0.04) | ns (p=0.07) |

**KI67**

|  | 1DIV | 1WIV | 6WIV |
| --- | --- | --- | --- |
| hNSC#1 vs. hNSC#2 | YES (***p<0.001) | YES (***p<0.001) | ns (p>0.99) |
| hNSC#1 vs. hNSC#3 | YES (***p<0.001) | YES (***p<0.001) | ns (p=0.91) |
| hNSC#1 vs. hNSC#4 | YES (***p<0.001) | YES (***p<0.001) | ns (p>0.99) |
| hNSC#2 vs. hNSC#3 | ns (p=0.91) | ns (p>0.99) | ns (p=0.84) |
| hNSC#2 vs. hNSC#4 | ns (p=0.14) | ns (p>0.99) | ns (p>0.99) |
| hNSC#3 vs. hNSC#4 | ns (p=0.42) | ns (p>0.99) | ns (p=0.90) |

|  | hNSC#1 | hNSC#2 | hNSC#3 | hNSC#4 |
| --- | --- | --- | --- | --- |
| 1DIV vs. 1WIV | YES (**p=0.003) | YES (*p=0.02) | ns (p=0.07) | ns (p=0.80) |
| 1DIV vs. 6WIV | YES (***p<0.001) | YES (**p=0.009) | ns (p=0.21) | ns (p=0.66) |
| 1WIV vs. 6WIV | YES (***p<0.001) | ns (p=0.95) | ns (p=0.81) | ns (p=0.97) |

**TUNEL ASSAY**

|  | 1DIV | 1WIV | 6WIV |
| --- | --- | --- | --- |
| hNSC#1 vs. hNSC#2 | ns (p=0.89) | ns (p=0.54) | YES (***p<0.001) |
| hNSC#1 vs. hNSC#3 | ns (p=0.99) | ns (p=0.83) | ns (p=0.94) |
| hNSC#1 vs. hNSC#4 | ns (p>0.99) | ns (p=0.73) | ns (p>0.99) |
| hNSC#2 vs. hNSC#3 | ns (p=0.98) | ns (p=0.96) | YES (***p<0.001) |
| hNSC#2 vs. hNSC#4 | ns (p=0.97) | ns (p=0.99) | YES (***p<0.001) |
| hNSC#3 vs. hNSC#4 | ns (p>0.99) | ns (p>0.99) | ns (p=0.96) |

|  | hNSC#1 | hNSC#2 | hNSC#3 | hNSC#4 |
| --- | --- | --- | --- | --- |
| 1DIV vs. 1WIV | ns (p=0.17) | ns (p=0.98) | ns (p=0.77) | ns (p=0.85) |
| 1DIV vs. 6WIV | YES (*p=0.01) | YES (***p<0.001) | YES (**p=0.007) | YES (*p=0.02) |
| 1WIV vs. 6WIV | ns (p=0.43) | YES (***p<0.001) | YES (*p=0.03) | ns (p=0.06) |

**TABLE S2** Detailed statistical analysis of comparisons between 2D-cultured hNSC on Cultrex and 3D-cultured hNSCs in HYDROSAP (two-way ANOVA followed by a BONFERRONI post-test). Significant differences (2D vs 3D) are marked with YES; ‘ns’ stands for not statistically significant; p is p-value.

|  | **βIII-Tubulin** | **GFAP** | **GALC/O4** | **NESTIN** |
| --- | --- | --- | --- | --- |
| **hNSC#1** | ns | YES (*p=0.01) | ns (p=0.21) | YES (**p=0.006) |
| **hNSC#2** | YES (*p=0.02) | ns (p=0.56) | ns (p>0.99) | ns (p>0.99 |
| **hNSC#3** | ns (p>0.99) | YES (**p=0.002) | ns (p>0.99) | YES (***p<0.001) |
| **hNSC#4** | YES (***p<0.001) | YES (**p=0.002) | YES (***p<0.001) | YES (**p=0.006) |
